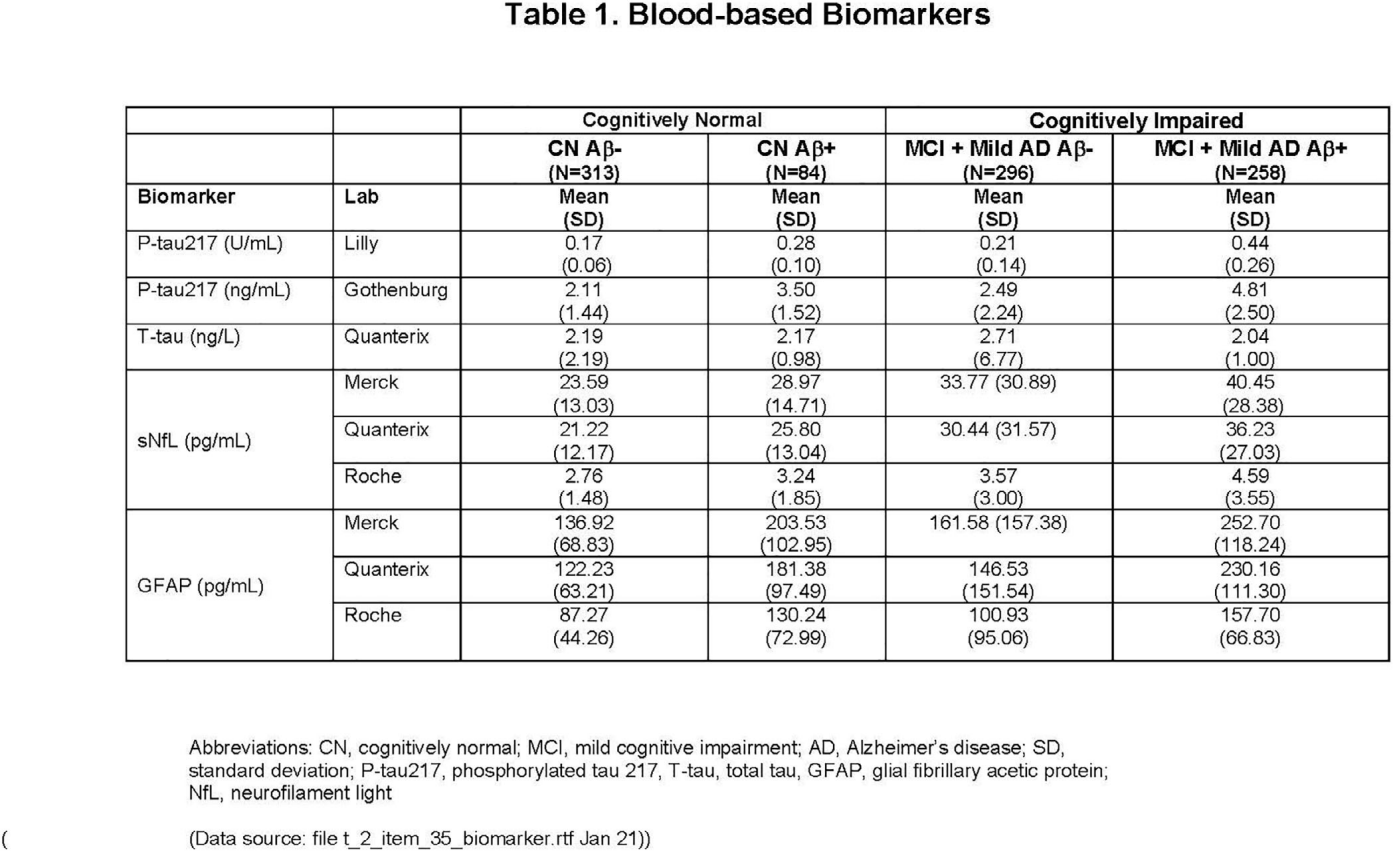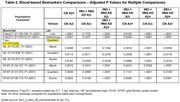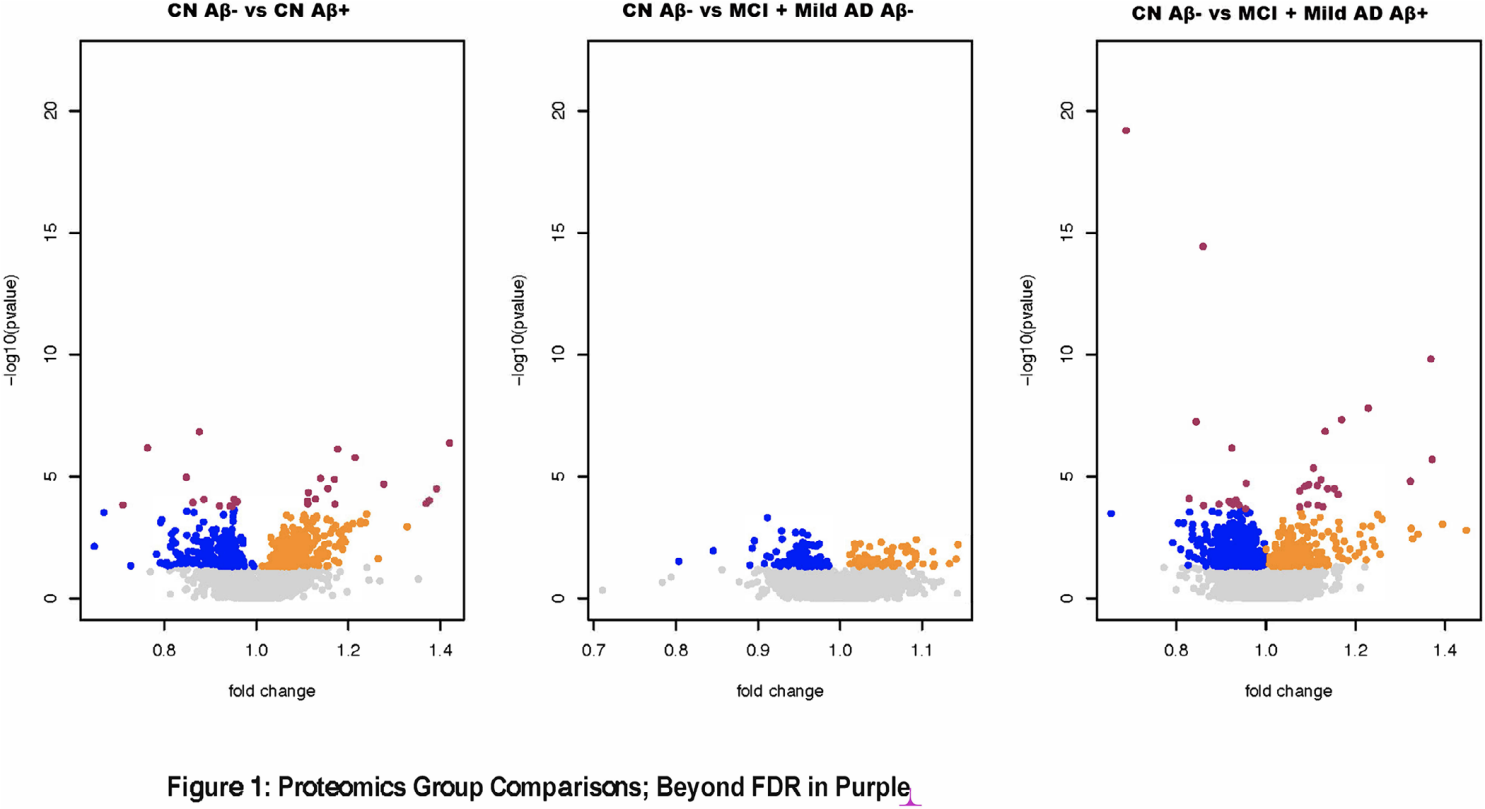# Biomarkers and proteomics in amyloid PET negative persons with clinical signs of MCI or mild dementia

**DOI:** 10.1002/alz70856_105032

**Published:** 2026-01-08

**Authors:** Richard Mohs, Douglas W. Beauregard, Allan I. Levey, Erik C.B. Johnson, Jessie Nicodemus‐Johnson, Robin Wolz, John Dwyer

**Affiliations:** ^1^ Global Alzheimer's Platform Foundation, Washington, DC, USA; ^2^ Emory University School of Medicine, Atlanta, GA, USA; ^3^ Goizueta Alzheimer's Disease Research Center, Atlanta, GA, USA; ^4^ Pentara Corporation, Salt Lake City, UT, USA; ^5^ IXICO, London, London, United Kingdom

## Abstract

**Background:**

Screening for clinical trials identifies persons with clinical symptoms of Mild Cognitive Impairment (MCI) or Alzheimer's disease (AD) but no amyloid on PET. We compared blood biomarkers and proteomics in these screen failures with cognitively normal, amyloid PET negative persons and with amyloid PET positive MCI or mild AD patients.

**Method:**

We analyzed data from 296 persons with clinical symptoms of MCI or mild AD from the Bio‐Hermes study who were amyloid PET negative (MCI + AD AB‐); we compared them with 313 Bio‐Hermes participants who were cognitively normal and amyloid PET negative (CN AB‐) and with 258 amyloid PET positive persons with clinical MCI or mild AD (MCI + AD AB+). Measures included regional amyloid PET, plasma A‐beta40 and 42, total tau, *p*‐tau 181, *p*‐tau 217, NfL, GFAP, a panel of 69 cytokines (Fireplex) and 7596 proteins from the Somalogic panel.

**Result:**

Relative to the CN AB‐ group, the MCI + AD AB‐ group were older, more cognitvely and functionally impaired, had slightly less education and were more likely to be non‐white; the groups did not differ in gender or APOE4 rates. The groups did not differ on amyloid SUVR, *p*‐tau 217, t‐tau or GFAP. NfL was higher in MCI + AD AB‐ persons compared with CN AB‐ persons (Table 1 and 2). After using a False Discovery Rate adjustment there were no Somalogic proteins significantly different in MCI + AD AB‐ participants vs CN AB‐ participants. Several proteins were different in amyloid PET positive persons compared with amyloid PET negative parsons (Figure 1).

**Conclusion:**

NfL data indicate very significant neurodegeneration in symptomatic but amyloid PET negative clinical trial screen failures. Biomarkers reflecting amyloid and tau were not different and the proteomics profile did not find specific abnormalities in amyloid negative, symptomatic persons.